# Enduring sex-dependent implications of pubertal stress on the gut-brain axis and mental health

**DOI:** 10.3389/fnbeh.2023.1285475

**Published:** 2024-01-11

**Authors:** Michaela Dworsky-Fried, Jessica A. Tchida, Rebecca Krnel, Nafissa Ismail

**Affiliations:** ^1^NISE Laboratory, School of Psychology, University of Ottawa, Ottawa, ON, Canada; ^2^University of Ottawa Brain and Mind Research Institute, Ottawa, ON, Canada; ^3^LIFE Research Institute, Ottawa, ON, Canada

**Keywords:** gut-brain axis, stress, adolescence, puberty, mood disorder, anxiety, brain, behavior hyperlink

## Abstract

The gut-brain axis (GBA) is a network responsible for the bidirectional communication between the central nervous system and the gastrointestinal tract. This multifaceted system is comprised of a complex microbiota, which may be altered by both intrinsic and extrinsic factors. During critical periods of development, these intrinsic and extrinsic factors can cause long-lasting sex-dependent changes in the GBA, which can affect brain structure and function. However, there is limited understanding of how the GBA is altered by stress and how it may be linked to the onset of mental illness during puberty. This article reviews current literature on the relationships between the GBA, the effects of stress during puberty, and the implications for mental health.

## Introduction

Throughout the lifespan, individuals undergo numerous critical periods of development during which maturation of the brain occurs, and heightened vulnerability to stressors is experienced (Sisk and Gee, 2022). Puberty is a stress-sensitive period, and exposure to stressful experiences during this time increases the susceptibility of enduring neural and behavioral effects (Murray et al., 2019). Stress, defined as the mental, emotional, and/or physical demands that extend beyond an individual’s regulatory capacity, has different impacts depending on the frequency, magnitude, and duration of the stress experience (Sheth et al., 2017). Puberty is also characterized by sexual maturation (Sisk and Foster, 2004). Physiological reproductive maturation begins with the activation of the hypothalamic–pituitary-gonadal (HPG) axis (Marceau et al., 2011; Tinggaard et al., 2012) following the binding of a hypothalamic peptide hormone, kisspeptin, to its receptor, Kiss1R. This binding stimulates gonadotropin-releasing hormone neurons to produce and release gonadotropin-releasing hormone (Dedes, 2012; Dagklis et al., 2015; Uenoyama et al., 2019; Xie et al., 2022) and stimulate the release of luteinizing and follicle-stimulating hormones. Luteinizing and follicle-stimulating hormones are released from the anterior pituitary gland into the bloodstream to initiate the production of gonadal steroid hormones in the testes and ovaries (Hiller-Sturmhöfel and Bartke, 1998). Once released, the increasing levels of gonadal hormones stimulate the development of secondary sex characteristics (Dedes, 2012; Dagklis et al., 2015), such as physical changes in body hair growth, fat deposition, and breast and genitalia development in females and males, respectively (Marshall and Tanner, 1969, 1970). In addition to these changes, evidence suggests that the HPG axis may play a role in neurodevelopment. A review of the literature conducted by Herting and Sowell (2017) found that the increasing concentrations of gonadal hormones during puberty are involved in both gray and white matter development, highlighting the significance of these gonadal hormones on brain maturation.

The timing of pubertal development has been found to differ between females and males. In humans, females typically undergo pubertal development between the ages of 8 and 13, whereas males undergo puberty between the ages of 9 and 14 (Marshall and Tanner, 1969, 1970; Farello et al., 2019). Several factors impact the timing of pubertal development. For example, there appears to be a link between pubertal timing and genetics for both females and males (Stroud and Davila, 2011; Wohlfahrt-Veje et al., 2016), as well as influences from socio-economic, demographic (e.g., place of residence, family type, first language learnt), and lifestyle factors (Al-Sahab et al., 2010; Stroud and Davila, 2011; Wohlfahrt-Veje et al., 2016). Given the involvement of the HPG axis in the onset of puberty, it provides further grounds for investigating the sex-specific effects of pubertal exposure to stressors and the ensuing mental health implications.

Pubertal development in mice is similar to that of humans (Blaustein et al., 2016). In female mice, puberty begins with vaginal opening as the first sign of ovarian activity at approximately postnatal (PND) day 30 (Kane and Ismail, 2017; Murray et al., 2023). In males, puberty typically begins with preputial separation around PND 42 (Kane and Ismail, 2017). The pubertal period is complete upon sperm production (around PND 50) in males and first estrus (around PND 60) in females (Smith et al., 2023). Factors, like strain, weaning age, and housing conditions impact the pubertal timing in mice (Bailoo et al., 2020). Like humans, mice also experience heightened vulnerability to stress during puberty. Specifically, exposure to pubertal stress can induce enduring alterations to the hypothalamic–pituitary–adrenal (HPA) axis, the brain, and behavior (Blaustein et al., 2016).

## The impacts of pubertal stress on the HPA axis

The HPA axis, known as the stress response system, also undergoes maturation during puberty (Smith et al., 2021). When encountered with a stressor, the HPA axis becomes activated, which initiates the stimulation and release of corticotropin-releasing hormone in the paraventricular nucleus of the hypothalamus (Herman et al., 2016). Corticotropin-releasing hormone then stimulates adrenocorticotropic hormone in the anterior pituitary gland before traveling through the bloodstream to the adrenal cortex. As a result, glucocorticoid stress hormones, most notably cortisol in humans or corticosterone in rodents, are released (Smith and Vale, 2006). Glucocorticoids negatively impact key brain regions responsible for regulating the HPA axis, such as the hippocampus, amygdala, and prefrontal cortex (Conrad, 2008; McCormick and Mathews, 2010), which could increase susceptibility to stress-related mental illnesses like depression and anxiety (Sinha, 2008; Packard et al., 2016). Alternatively, repeated exposure to stressor during puberty can result in the blunting of the HPA axis (Trickett et al., 2010). Pubertal stress exposure may increase the expression of glucocorticoid receptors, facilitating the downregulation of glucocorticoids and the blunting of the HPA axis (Susman, 2006). Consequently, the blunting of the HPA axis could increase susceptibility to disorders such as post-traumatic stress disorder and personality disorders (Cohen et al., 2006; Fairchild et al., 2018; Drews et al., 2019). Additionally, exposure to an immune challenge during critical neurodevelopmental periods can produce a “programming” effect when encountering stress later in life. This effect occurs when a specific environmental factor is experienced during development and influences responses to subsequent stress exposure. Programming effects can lead to diseases because of a prolonged immune response, primarily when stress exposure occurs during puberty (Sharma et al., 2019).

Earlier studies using mice and rats have examined the effects of various pubertal stressors, such as restraint (Klinger et al., 2019), forced swim (Wilkin et al., 2012), foot-shock (Liu et al., 2017), and social isolation stress (Lukkes et al., 2009) on the behavior adults. Exposure to these stressors during puberty caused enduring anxiety- and depression-like behaviors (Pellow et al., 1985; Carola et al., 2002), and these effects appear to be sex-dependent. For instance, exposure to social stress during puberty induces anxiety-like behavior in both sexes, but it induces depression-like behavior in females only (Caruso et al., 2017). Similarly, exposure to a single injection of LPS during puberty causes enduring depression-like behavior in female mice and anxiety-like behavior in male mice (Murray et al., 2019). These findings highlight that stress-induced impacts on mental health are sex-dependent and provide a fundamental basis for the need for specialized interventions.

The mechanisms underlying the observed sex differences in stress responses remain to be fully elucidated. One likely factor is the influence of gonadal hormones on the HPA axis. In rodents, estrogen and testosterone distinctly modulate corticosterone levels, suggesting an interaction between the HPA and HPG axes (McCormick and Mathews, 2010). Differences in corticosterone release from the adrenal gland between male and female rodents become especially pronounced after exposure to stress. For example, female rats show higher corticosterone levels that persist for a more extended period than those in males following stress (Kant et al., 1983; Seale et al., 2004; Figueiredo et al., 2007). This difference is often attributed to the role of estrogens, particularly estradiol, in modulating glucocorticoid responses in females. Estradiol levels peak during the proestrus phase of the estrous cycle (Oyola and Handa, 2017). As a result, female mice in proestrus show heightened anxiety-like behaviors in response to stressors compared to females in estrus (McCormick et al., 2008; Lovick and Zangrossi, 2021).

In contrast, in male rodents, testosterone attenuates the HPA axis response to stress and induces anxiolytic-like effects via androgen receptors (Bernardi et al., 1989; Edinger and Frye, 2005; Chen et al., 2014). Gonadectomies in animal models highlight the influence of heightened gonadal hormones at puberty on behavior. Boivin et al. (2017) found that pre-pubertal gonadectomy increased anxiety-like behavior in males, but not in females, during adolescence. Another study found significant differences between adult male and female mice gonadectomized before pubertal onset. Gonadectomized males display increased anxiety-like behavior, while gonadectomized females show the contrary (Delevich et al., 2020). Similarly, in boys between 14 and 17 years old, lower testosterone levels are associated with increased symptoms of anxiety (Granger et al., 2003). Moreover, males with hypogonadism, a condition characterized by impaired gonadal function and reduced testosterone levels (McHenry et al., 2014), are more prone to anxiety and major depressive disorders than those with normal androgen levels (Zarrouf et al., 2009).

## The gut-brain axis

**Figure 1:**
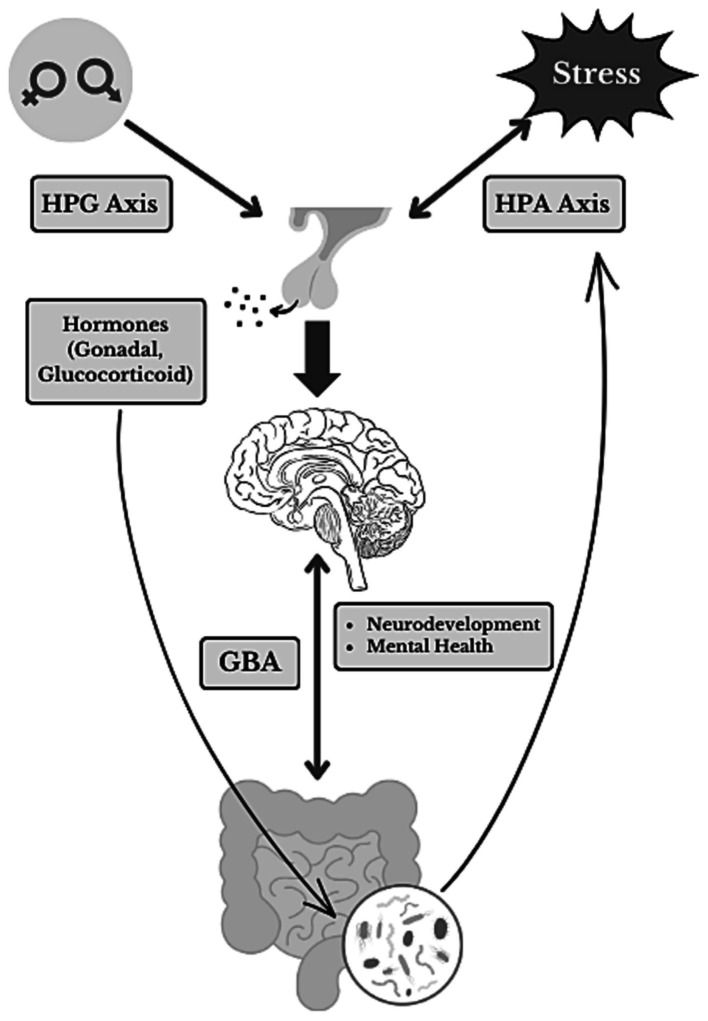
Sex-mediated and stress-induced GBA interactions. A summary diagram illustrating the complex relationships within the gut-brain axis (GBA) as well as the influence of pubertal stress-exposure on neurodevelopment and mental health. The diagram showcases the interconnected elements of sex, stress, critical neuroendocrine pathways [hypothalamic–pituitary-gonadal (HPG) and hypothalamic–pituitary–adrenal (HPA) axes] and hormones, and the gut microbiome. Directional arrows illustrate the flow of information and key interactions.

Emerging research suggests that the gut microbiome might play a role in the sex-specific effects of pubertal stress on the brain and behavior (see Figure 1; Cryan and Dinan, 2012; Murray et al., 2019, 2020). The gut microbiome, consisting of trillions of bacteria, peptides, fungi, and viruses, impacts an individual’s health through metabolic, immune, and physiological functions (Marchesi and Ravel, 2015; Shreiner et al., 2015; Bauer et al., 2016). The gut-brain axis (GBA) is a bidirectional network between the central nervous system, the autonomic nervous system, the enteric nervous system, and the HPA axis (Mayer, 2011). The GBA establishes a link between the brain regions involved in cognitive function and emotional regulation and intestinal functions (Carabotti et al., 2015), exemplified by studies involving germ-free (GF) rodents devoid of gut microbiota from birth. GF mice exhibit distinct dendritic morphological changes in the amygdala and hippocampus, impacting emotional regulation, memory and learning, and neuroplasticity (Luczynski et al., 2016). Additionally, GF rats displayed exacerbated neuroendocrine and behavioral responses to acute stress. Specifically, increased anxiety-like behavior and an exacerbated HPA axis response, reflected by increased corticosterone levels, were observed compared to controls (Crumeyrolle-Arias et al., 2014).

Furthermore, animal studies demonstrate sex differences in gut microbiota composition (Yuan et al., 2020; Sisk-Hackworth et al., 2023). For example, Yurkovetskiy et al. (2013) found that the *Porphyromonadaceae, Veillonellaceae, Kineosporiaceae, Peptococcaceae, Enterobacteriaceae, Lactobacillaceae, Cytophagaceae, Peptostreptococcaceae, and Bacteroidaceae* families are more abundant in males than in female mice. Gonadal hormones during puberty play a critical role in mediating these sex differences in gut microbiota composition. Male mice castrated at 6 weeks of age (during puberty) display a microbiota composition that is more similar to that of female mice than non-castrated males (Yurkovetskiy et al., 2013). However, testosterone treatment following pubertal castration mitigated the difference in gut microbiota composition castrated and intact males (Org et al., 2016).

The gut microbiome is also influenced by estrogens via the estrobolome, a collection of bacteria in the gut capable of modulating the concentrations of estrogens in the body (Kwa et al., 2016). Like testosterone, estradiol can also modulate the gut microbiota. Ovariectomy induces a shift in the ratio of gut microbiome phyla, increasing the abundance of Firmicutes compared to Bacteroidetes (Acharya et al., 2023). A high abundance of Firmicutes compared to Bacteroidetes is associated with metabolic disorders (Moreno-Indias et al., 2016). For example, sex differences in multiple sclerosis (MS) were associated with estradiol-mediated changes in the gut microbiome. Specifically, estradiol treatment in mice prevented MS in males and ovariectomized females by altering the gut microbiome and intestinal alkaline phosphatase, an enzyme crucial in regulating gut microbes (Kaliannan et al., 2018).

## Link between the gut microbiome and mental health

The surge of research in the gut microbiome has revealed that disruptions of gastrointestinal microbiota play a critical role in the development of mental disorders. Specifically, gut dysbiosis – an “imbalance” in the gut microbial community (Johnson, 2006) – is associated with depression and anxiety through the GBA, by activating the immune system or altering communication between the gut and the brain (Rathour et al., 2022). The involvement of the HPA axis in the GBA illustrates the role that stress plays in this interaction. Notably, stressors such as the bacterial endotoxin lipopolysaccharide (LPS) have been shown to activate the immune system and cause disturbances to the gut microbiome (Cani et al., 2008) and behavior in an age- and sex-dependent manner in mice (Cai et al., 2016; Sharma et al., 2018). Interestingly, LPS treatment induces more pronounced changes in the microbiota composition of males and females during puberty than during adulthood. Moreover, male mice show greater depletion of gut microbiota diversity and increased anxiety-like behaviors than females following pubertal LPS exposure (Murray et al., 2020). This line of research underscores the intricate connections between immune system activation, gut microbiome disturbances, and behavioral changes, and highlights the complex interplay between gut dysbiosis and mental health.

Fecal microbiota transplantation (FMT) from adult mice with gut dysbiosis to GF mice showed increased depression-like behavior in GF mice compared to controls (Bruce-Keller et al., 2015; Zheng et al., 2016), illustrating the influence of alterations to the gut microbiome on depression. Furthermore, mice transplanted with microbiota from donor mice, exposed to chronic unpredictable stress, exhibited increased anxiety- and depression-like behaviors, reduced abundance of *Lactobacillus* and increased *Akkermansia* in the gut microbiota, and elevated pro-inflammatory cytokines in the brain (Li et al., 2019). Further research considering sex differences and the effectiveness of FMT for mental illnesses could significantly transform existing approaches to mental health treatment.

Founded by pre-clinical studies, novel treatment avenues in clinical settings have been recently explored for depression based on the intricate relationship between gastrointestinal and mental illnesses. In 2022, two adult patients with major depressive disorder (MDD) and gastrointestinal (GI) symptoms like constipation were treated with FMT in conjunction with pharmacological and psychological interventions. The patients were administered 30 oral capsules, and within 4 weeks of the FMT, both patients displayed improved depressive and GI symptoms (Doll et al., 2022). Another study used FMT in three adult participants with MDD and a history of irritable bowel syndrome via an enema and colonoscopy infusions. Within the following 6 months, the participants experienced significantly improved GI symptoms and an overall reduction in symptoms of depression (Collyer et al., 2020). Although it is unclear whether these patients have a history of pubertal stress, these findings echo earlier research indicating that disruptions in the gut microbiome can influence healthy brain functioning. These studies underscore the critical need to continue conducting clinical trials to establish if FMT can provide enduring improvements in depression with safe and controlled methods.

The overall health and resiliency of the gut microbiome are highly dependent on the diversity and richness of microbiota composition. Consequently, probiotics have been commonly used as a potential vehicle to restore disturbed microbiota and affect mood by regulating the GBA. Increasing evidence shows that exposure to a probiotic-supplemented diet can mitigate the effects of LPS-induced microbial changes. For example, exposure to Kefir containing active bacterial culture (*L. lactis, L. cremoris, L. diacetylactis, L. acid-ophilus*), lactic yeasts and skim milk powder one-week prior to and one-week following pubertal LPS treatment reduced sickness behavior (Murray et al., 2019). Another study using a probiotic mixture containing *Lactobacillus helveticus* and *Bifidobacterium longum* showed decreased cytokine concentrations in the blood of CD-1 mice (Esposito et al., 2022). These findings hold significant implications, as elevated levels of inflammatory cytokines are known to be associated with an increased risk of neuropsychiatric disorders and depression (Felger and Lotrich, 2013). Moreover, probiotics have shown to hinder LPS-induced enduring negative effects on both anxiety and depression-like behaviors (Murray et al., 2019, 2020) compared to controls in mice. Animal studies have also found that probiotic treatment prevented stress-induced increases in adrenocorticotropic hormone, corticosterone, adrenaline, and noradrenaline (Ait-Belgnaoui et al., 2012, 2014). The reduction in these stress hormones suggests probiotics attenuated the HPA axis, which is hyperactive in individuals with depression (Keller et al., 2017). Additionally, probiotics have been shown to increase the expression of brain-derived neurotrophic factor, a growth factor typically reduced in depressed individuals (Sen et al., 2008). These findings suggest that probiotics have a positive impact on the central nervous system by regulating critical neurotransmitters involved in depression.

## Conclusions and future directions

The findings presented in this review shed light on the relationship between stressful events during puberty and their impact on gut disturbances and increased vulnerability to mental health. The journey of unraveling the intricate mechanisms underlying mental illnesses, including depression and anxiety, has been significantly advanced through continuous research into the GBA. Pre-clinical models and preliminary clinical research support dysbiosis’s role in several psychiatric conditions. However, several questions remain unanswered, and certain limitations persist. Few clinical studies in this field have been published and are often small-scale (Dinan and Cryan, 2017). Therefore, identifying suitable animal models becomes paramount in developing generalizable methodologies within this domain. Considering factors such as sex, age, strain, and housing conditions of the mice used in these models will be crucial for obtaining relevant and meaningful results. Despite the numerous advantages of animal models and the significant similarities to humans, mice differ genetically, anatomically, and physiologically. Thus, non-human models can only partially embody the human gut microbiome.

Sex differences play a significant role in human health. Many illnesses, including diseases of the immune system and mental illnesses, display sex-dependent differences in prevalence, presentation, and response to treatment. The inadequate focus on sex differences in animal models leads to an inaccurate representation of human diseases, posing the risk of misinterpretations and false generalizations. Researchers must study these differences in animal models to gain insight into the underlying mechanisms and potential treatment avenues that might be more effective for specific sexes. Furthermore, sex differences can be influenced by hormonal factors. One confounding factor not often considered is the estrous cycle in female mice. Females in proestrus or estrus phases are less anxious than females in diestrus or metestrus (Rodríguez-Landa et al., 2021) and less anxious than males (Kokras et al., 2012). Consequently, it is important to consider the estrous cycle of female mice when testing anxiety-like behaviors.

Future studies should delve deeper into the potential of probiotics and FMT as promising therapeutic and preventive options for individuals struggling with mental health issues. However, to fully acknowledge and accept such advancements, rigorous research using appropriate experimental designs must validate their efficacy and safety. A key challenge of using probiotics is understanding the possible interactions of the probiotics with host cells and their respective safe doses. Improved studies are necessary to establish the use of probiotics more effectively and in the proper quantities. This will ensure that probiotics can be harnessed safely as a complementary approach to existing treatments for mental illnesses. Moreover, studies often do not adopt a longitudinal approach to explore the relationship between probiotics and the long-term shifts to the microbiome following stress. This approach in future studies is valuable to help understand changes likely to occur in humans. Similarly, more research is needed to better understand the key factors involved in the efficacy and use of FMT treatments. Currently, it is unclear which properties of a transplanted sample contribute to the therapeutic effects and whether any of the non-microbial components of donor stool are necessary to obtain successful results (Wilson et al., 2019; Lavelle and Sokol, 2022). Any differences in the effectiveness of the various possible methods of administration are also poorly understood (Lavelle and Sokol, 2022). Determining the ideal microbial profile of the donor sample and method of administration will be crucial in improving clinical outcomes.

In conclusion, the link between stressful experiences during puberty, gut disturbances, and mental health vulnerability offers a promising avenue for research opportunities. By continually exploring the GBA and its mechanisms, we can gain valuable insights into the development and potential treatment of mental illnesses. Addressing the existing knowledge gaps and limitations is paramount to comprehensively understanding of this complex relationship. By doing so, we pave the way for innovative therapies and interventions that could significantly improve the lives of those affected by mental health challenges.

## Author contributions

MD-F: Writing – original draft, Writing – review & editing. JT: Conceptualization, Writing – original draft. RK: Writing – review & editing. NI: Conceptualization, Funding acquisition, Methodology, Project administration, Resources, Supervision, Visualization, Writing – review & editing.
